# The “Fortilat” Randomized Clinical Trial Follow-Up: Auxological Outcome at 18 Months of Age

**DOI:** 10.3390/nu12123730

**Published:** 2020-12-03

**Authors:** Chiara Peila, Elena Spada, Enrico Bertino, Sonia Deantoni, Federica Percivati, Guido E. Moro, Marzia Giribaldi, Laura Cavallarin, Francesco Cresi, Alessandra Coscia

**Affiliations:** 1 Department of Public Health and Pediatric, Neonatal Unit of Turin University, Via Ventimiglia 3, 10126 Turin, Italy; elenaspada.bios@gmail.com (E.S.); enrico.bertino@unito.it (E.B.); deantoni.so09@gmail.com (S.D.); federica.percivati@edu.unito.it (F.P.); francesco.cresi@unito.it (F.C.); alessandra.coscia@unito.it (A.C.); 2 Italian Association of Human Milk Banks, Via Libero Temolo 4, 20126 Milan, Italy; guidoemoro@tiscali.it; 3 Institute of Sciences of Food Production, National Research Council, Largo Braccini 2, 10095 Grugliasco, Italy; marzia.giribaldi@ispa.cnr.it (M.G.); laura.cavallarin@ispa.cnr.it (L.C.); 4 Research Centre for Engineering and Agro-Food Processing, Council for Agricultural Research and Economics (CREA), Strada delle Cacce 73, 10135 Turin, Italy

**Keywords:** human milk, human milk fortifier, donkey milk, adjustable fortification, VLBW infants, preterm infants, catch-up growth, auxological outcomes

## Abstract

Human milk fortification is a routine clinical practice for feeding preterm infants. We hypothesized that donkey milk can be a suitable basis for developing an innovative human milk fortifier. Our randomized controlled single-blind clinical trial, named “Fortilat”, evaluated the feeding tolerance, growth and clinical short-term outcomes in a population of preterm infants fed with a novel multi-component fortifier and a protein concentrate derived from donkey milk. The aim of the current study is to extend the previous findings and to evaluate the auxological outcomes of the infants enrolled in the “Fortilat” trial at 18 months of age. In the previous trial “Fortilat”, the fortification protocol followed was the same for the two groups, and the two diets were designed to be isoproteic and isocaloric. All infants enrolled in the trial were included in a premature infant developmental evaluation program consisting of hospital visits at 40 ± 1 weeks of postmenstrual age, and at 6, 12 and 18 months of corrected age. Weight, head circumference and length were expressed in z-score using neonatal Intergrowth21st and INeS charts at birth, and WHO 0–5 years growth charts at 18 months. 122 children (Bovine-arm = 62, Donkey-arm = 60) were included in this study. All the observations were recorded in the interval of 18 ± 3 months of the correct age. The two groups did not differ for head circumference, length or weight at 18 months of age. Our data show that fortifiers derived from donkey milk had not different long term auxological outcomes of standard bovine-derived fortifier, but the new donkey milk fortifier was well tolerated in our population.

## 1. Introduction

Very preterm newborns (gestational age < 32 weeks) and Very Low Birthweight infants (VLBW, birthweight < 1500 g) represent the majority of patients assisted in Neonatal Intensive Care Units (NICU) [1]. The increase of the survival rate for these newborns, due to improvements in perinatal care, has opened new perspectives regarding their outcome and their health status in adulthood.

Nutrition represents a fundamental factor for long-term postnatal growth and quality of life, including potential influence on positive neurocognitive outcomes for VLBW infants [2,3]. In this context, the European Milk Banks Association (EMBA) highlights that achieving optimal growth and adequate nutrition are the main targets for the successful management of preterm infant care [4,5]. Some studies also suggested that inadequate nutritional management for preterm infants may increase the risk of developing cardiovascular and metabolic diseases in adult life, although evidence is still low at present [6].

The main issue in clinical practice is to ensure adequate qualitative and quantitative nutrition, particularly in terms of protein intake which is the main cause of post-natal growth deficit [4]. Thus, providing optimal nutrition in the neonatal period, particularly for VLBW infants, has become a priority for reducing short- and long-term complications. Indeed, several investigations focused on neonatal nutrition, post-discharge growth and development pointed out that: (i) better nutrition is associated with improved growth [7,8]; (ii) improved growth is associated with improved neurodevelopmental outcomes [9,10]; (iii) better nutrition is associated with improved neurodevelopmental outcomes [11,12]. Antenatal and postnatal growth, especially that recorded in head circumference, are associated with long-term neurodevelopmental outcomes [13].

Although human milk (HM) is undoubtedly the gold standard of nutrition for every newborn, in case of premature birth, it is inadequate for the nutritional needs of infants since it provides insufficient amounts of several nutrients [14,15,16]. HM must therefore be fortified with the nutrients in short supply [4,14,17,18]. Although HM fortification is widely adopted in NICUs worldwide, preterm infants that received the fortification often still experience suboptimal growth and feeding intolerance. Thus, during the last decade, new fortification strategies and different commercially available fortifiers have been developed and tested. Nevertheless, the optimal method for HM fortification remains to be determined, and a variety of protocols are currently used [4,19,20,21,22,23]. Among these, Adjustable (ADJ) Fortification, an “Individualized Fortification’’ method, currently seems to be the most promising [4]. It involves the use of a protein supplement on an individualized basis, in addition to a multi-component fortifier. This method, accepted at an international level, consists of providing a variable protein intake based on the metabolic response of every single newborn by titration to the infant’s blood urea nitrogen level [4,21,23].

In this contest, our group recently conducted a randomized clinical trial named “Fortilat”, which aimed to compare the effects of a bovine milk-based fortifier (BF) with an experimental donkey milk-based (DF) fortifier [24,25]. Since donkey milk has a protein profile more similar to that of human milk in terms of relative abundance and primary structure in comparison with bovine milk [26], we hypothesized that such differences may impact the protein utilization in preterm infants and, consequently, that donkey milk is more suitable than bovine milk as an ingredient in human milk fortifiers. Our data showed that preterm and VLBW infants receiving isocaloric and isoproteic supplementation of HM (according to ADJ fortification protocol) with either BF or DF, achieved similar mean daily weight growth between the first day of exclusive enteral feeding and discharge. Moreover, DF reduced the occurrence of episodes of feeding intolerance, feeding interruptions, bilious gastric residuals and vomiting [25]. In an ancillary study, the use of DF reduced also the gastroesophageal reflux frequency in infants showing infants with clinical signs of gastroesophageal reflux and cardiorespiratory symptoms associated with feeding intolerance [27]. A recent urinary metabolomics investigation revealed that the different quality of the nutrients provided by isocaloric and isoproteic fortification with either DF and BF resulted in different urinary metabolic patterns [28].

The aim of the current study is to extend these previous findings and to evaluate the auxological outcomes at 18 months of age of the “Fortilat” trial.

## 2. Materials and Methods

### 2.1. Clinical Trial and Intervention

The study was performed in the NICU of the University, City of Health and Science of Turin, was approved by Local Ethic Committee (AN: 0025847, 27 May 2014), and registered (ISRCTN70022881). Recruitment period was between 27 November 2014 to 22 December 2016.

The inclusion criteria were: gestational age < 32 weeks or birthweight < 1501 g, exclusive feeding with HM (fresh own mother’s or donor milk) and enteral feeding ≥ 80mL/kg/day of HM reached within the first 4 weeks of life. Neonates affected by severe gastrointestinal pathologies (such as necrotizing enterocolitis, colostomy, intestinal obstruction, symptoms of peritonitis, presence of blood in the feces), chromosomal abnormalities or major malformations, hereditary metabolic diseases, intravascular disseminated coagulopathy (IDC), shock, patent ductus arteriosus (PDA) requiring medical care or surgery at time of randomization, or severe renal failure (serum creatinine > 2 mg/dL) were excluded. After informed written parental consent was obtained, infants were randomized 1:1 by a software-generated list in one of the following groups: the control group (BF-arm) underwent ADJ fortification with a multi-component fortifier (FM85—Nestlè) and a protein concentrate (Protifar—Nutricia) derived from bovine milk; the Fortilat-group (DF-arm) underwent ADJ fortification with a multi-component fortifier and a protein concentrate derived from donkey milk. Please refer to our previous papers for a detailed description of the study protocol, including characteristics of the protein supplements used in the trial [24]. Briefly, the experimental products were produced by ultrafiltration of pasteurized donkey milk in a pilot stainless steel plant. Retentates from the ultrafiltration processes were then pasteurized and aseptically lyophilized and packed. All the batches used for the trial were analysed for the microbiological and chemical profile and complied with the safety criteria required by Italian legislation. The products were stored at −80 °C until used.

### 2.2. The Fortilat Follow-Up

All infants enrolled in the trial were included in a premature infant developmental assessment program that consisted of hospital visits at 40 ± 1 weeks of postmenstrual age, and at 6, 12 and 18 months of corrected age. At each visit, medical history taking, and growth evaluation were performed. Physical and neurological examinations were performed by an experienced neonatologist in the follow-up program. Regarding auxological parameters, weight, length and head circumference measurements were taken and recorded according to standard anthropometric procedures. The neonatologists took measurements using identical equipment: an electronic scale (Seca, Hangzhou, China) for weight, a specially designed Harpenden infantometer (Chasmors, London, UK) for length, and a metallic non-extendable tape (Chasmors) for head circumference. The equipment, which was calibrated twice a month, was selected for accuracy, precision, and robustness. Measurement procedures were standardized on the basis of WHO recommendations to ensure maximum validity [29,30].

### 2.3. Subjects and Statistical Analysis

The randomized clinical trial included 156 subjects: BF-arm *n* = 79, DF-arm *n* = 77. In the present investigation, we considered only children whose auxological information at 18 months of age were available. Weight, head circumference and length were expressed in z-score using neonatal Intergrowth21st [31] and INeS charts [32] at birth, and WHO 0–5 years’ growth charts [https://www.who.int/childgrowth/standards/en/] at 18 months. Z-score values lower than −4 or higher than +4 were considered outliers. The probability of observing a z-score with a value out of the range −4, +4 is 0.006% in the target population. Even if we expected our babies are smaller than target population babies, it is plausible that a z-score lower than −4 or higher than +4 is an outlier. The children with birth weight lower than the 10th or higher than the 90th centile were defined as small (SGA) or Large (LGA) for gestational age (GA), respectively. The categorical variables were summarized as absolute frequencies (percent); the continuous variables were summarized as mean (SD) or media [Inter Quartile Range] plus (minimum–maximum) according to their distribution. The difference in z-score at 18 months between the two arms of the trial was estimated using the T-test on the assumption of casual loss at follow-up. A further analysis was performed using generalized linear models including arm, z-scores at birth (according to neonatal Intergrowth21st or INeS charts), GA at birth and segmented on population (GA at birth < 32 weeks, GA at birth ≥ 32 weeks with birth weight < 1501 g) as covariates. The last covariate was included because the double inclusion criteria (all neonates with GA < 32 weeks and neonates with GA ≥ 32 weeks only if birth weight is less than 1501 g) defined two different groups (or populations) of neonates. The first population (GA < 32 weeks) included neonates with a higher risk of morbidities related to low GA, whereas the second population included a higher proportion of females and twins (both physiologically smaller), intrauterine growth restriction (IUGR) babies (pathologically smaller), and SGA babies (smaller by definition). Data analysis was performed with SAS^®^ software version 9.4 (Copyright © 2020 by SAS Institute Inc., Cary, NC, USA).

## 3. Results

Of the 156 children included in the original trial [23], 34 (17 per arm) were excluded, due to the lack of auxological data record at 18 months of age. One value for weight, one for length and one for head circumference, all at 18 months, were identified as outliers, and as such were not included in the statistical analysis. At birth, one baby had length z-score value of −4.04 according to Intergrowth21st neonatal charts, but it was not considered as outlier since the z-score was of −3.31 according to INeS charts.

Table 1 shows the basal characteristics of the 122 children (BF-arm = 62, DF-arm = 60) included in this study. The BF arm had 14% more neonates born GA < 32 weeks than the DF arm. This difference leads to a lower average gestational age (about 8 days), and to higher head circumference, length and weight mean z-scores at birth in the BF-arm with respect to the DF-arm.

All the observations recorded at 18 ± 3 months of correct age were included in the analysis. The median [InterQuartile Range, IQR] corrected age at the visit of auxological follow-up is 18 [17,18] months in BF-arm and 18 [17,18,19] months in DF-arm, while the chronological postnatal age is 20 [20,21] and 20 [19,20,21] months, respectively. Table 2 and Table 3 report the auxological traits z-score comparison between DF- and BF-arm at follow-up, estimated using T-test (Table 2) and general linear model (Table 3). In both approaches, the two groups did not differ for head circumference, length or weight at 18 months of age. On average, the children of this study are significantly shorter and lighter than the target population of WHO charts (mean z-score lower than and CI(95%) not including 0), while they do not differ in head circumference.

Table 4 reports the effect of GA, auxological trait z-score at birth, and Population (<32 weeks vs. ≥32 weeks) on average z-scores (for the same auxological variable) at 18 months of corrected age. The Population was not associated with the auxological variables at 18 months. GA was not significant for head circumference and had an effect between +0.1 (for weight) and about +0.2 (for length). This means that, while having the same z-score at birth and being part of the same population and arm, the expected z score at 18 months increased between 0.1 (weight) and 0.2 (length) at 18 months for an increment of 1 week in GA. Concerning the influence of z-scores at birth, the expected values in z-score at 18 months while having the same GA at birth and being part of the same population and arm, increased between 0.5 (for weight) and 0.6 (for length) for an increment of 1 z-score at birth. The estimates of the models are very similar considering z-score values according to neonatal Intergrowth21st or INeS charts.

## 4. Discussion

The first aim of the “Fortilat” study was to assess the effects of feeding donkey milk-derived human milk fortifier on feeding tolerance among very preterm newborns (gestational age < 32 weeks) and VLBW infants (<1501 g). All newborns (in both the BF-arm and the DF-arm) exclusively received HM (raw own mother’s milk or pasteurized donor milk), without any preterm formula supplementation. We have previously demonstrated that DF is better tolerated. Feeding DF reduced the occurrence of episodes of feeding intolerance, feeding interruptions, episodes of bilious gastric residuals and vomiting, in comparison to the bovine counterpart [25]. We speculated that the quality of donkey milk proteins could be responsible for this finding, the two diets being isoproteic and isocaloric, although differing in origin (bovine vs. donkey milk) and form (extensively hydrolyzed bovine whey proteins vs. whole donkey milk proteins). Major differences in the two diets included the type of carbohydrate (maltodextrins vs. lactose), and the type and quantity of lipids. Moreover, in a previous paper [25] we reported that the weight gain during hospitalization was similar in BF-and DF-arms, suggesting that differences in tolerance do not affect short-term growth. Milk from monogastric animals, rather than from ruminants, has been suggested during recent years to be more suitable for human nutrition based on its physicochemical properties, including a more similar protein and lipid composition to that of human milk [26,33]. In addition, it has been demonstrated in murine models that supplementation of the basal diet with donkey milk decreases the accumulation of body lipids and affects glucose and lipid metabolism in a manner more similar to human milk than cow milk [34].

In this expanded analysis of the follow-up data, we observed that DF- and BF- arms do not show a different pattern of growth in weight, length and head circumference also at 18 months of age of corrected age.

A limitation of the study is that after discharge all infants used bovine-derived standard fortifiers. On the other hand, all the infants received only human milk (mother’s own milk or Pasteurized donor milk) without any preterm formula supplementation to avoid confounding factors masking the effects of the fortifiers, and we observed the effects of the different fortification on long term growth throughout the hospital stay.

Notwithstanding the difference in composition between raw human milk and pasteurized human milk, we did not consider it necessary to report the results for the different study groups separately; the reason is that we applied an internationally accepted Individualized Fortification method (the ADJ Fortification).

Another limitation is that the study protocol was designed with a different aim (i.e., feeding intolerance during the observational period) and long-term growth was a secondary outcome.

On average, our study population is shorter and lighter than the WHO target population at the same age. Similar findings were reported in the literature [35,36,37]. The present findings confirm difficulties in meeting the optimal target for catch-up growth, which can be only partially achieved. On the other hand, the head circumference z-scores achieved mean values close to the median, thus indicating that the fortification protocol was successful in achieving such target growth. This finding can be interpreted by observing the WHO charts 0–5 years: while head circumference growth is faster during the first year of life with important consequences for related neurocognitive outcomes, weight and length have a strong slope of growth even after 2 years of age.

We found that different types of fortification do not have any effect not only on the short-term weight gain but also on the long term auxological outcomes for all anthropometric variables. This evidence further supports the validity of the fortifier derived from donkey milk.

It is worth noting that the two arms achieved similar growth at 18 months, even when considering that at the birth head circumference, length and weight mean z-scores were lower in DF-arm than BF-arm. This difference is probably due to the double inclusion criteria: in the DF-arm the percentage of newborns with GA ≥ 32 weeks was higher than in BF-arm. These babies were included in the trial when their birth weight was below 1501 g, consequently, they are smaller, but older by definition. In such a population, catch-up growth (physiological effect) and regression to the mean (statistical effect) are more likely to be observed [38,39,40]. For these reasons, sampling only for GA, or considering the population as a covariate in the analysis, would be an interesting alternative.

Considering the present data in relation to the other variables evaluated during our analysis, further considerations can be made. The size at birth was associated with the dimension at 18 months, while GA had a minor effect. We have to consider that in our population all newborns were preterm or VLBW infants. The population had no effect, but it could be considered as an important covariate because GA and auxological variables at birth were not uniform in the two populations by definition.

Regarding the two different neonatal charts used for the analysis, the differences in results were negligible. This indicates that the two charts are interchangeable in this kind of study. Since INeS charts have a major range of GA, this could imply losing fewer data at birth.

## 5. Conclusions

Our previous observations have shown that donkey milk-derived fortifiers improve the feeding tolerance in preterm infants when compared with standard bovine-derived fortifiers, with similar short-term auxological outcomes. Results from the evaluation of auxological outcomes at 18 months of corrected age demonstrate that donkey milk-derived fortifiers are at least as effective as commercial bovine milk-based fortifiers in supporting long-term growth, almost reaching the target growth for head circumference. The present findings are of significance, especially when considering that infants receiving donkey milk-derived fortifiers were meanly smaller at birth than those receiving bovine milk-derived commercial fortifiers. These results may constitute a basis on which to plan further trials aimed at confirming efficacy and higher tolerability of fortifiers based on donkey milk. This study constitutes the base for future research specifically planned with the aim of comparing the growth and its shape between the two different fortifiers.

## Figures and Tables

**Table 1:** Basal characteristics of the 122 children included in the study.

		BF-Arm *n* = 62	DF-Arm *n* = 60
Boys	*n* (%)	29 (46.8)	30 (50.0)
GA < 32 weeks	*n* (%)	52 (83.9)	42 (70.0)
GA (days)	median [IQR] (min–max)	209 (193–217] (153−238)	217 [206–225.5] (168−255)
**Birth head circumference** *			
cm	mean (SD) (min–max)	26.3 (2.3) (21.5–30.8)	27.0 (2.1) (20.0–30.1)
z-score Int21s	mean (SD) (min–max)	−0.52 (0.99) (−3.30–+1.49)	−0.91 (1.16) (−2.98–+1.34)
<10th centile Int21st	*n* (%)	15 (28.3)	21 (40.4)
>90th centile Int21st	*n* (%)	1 (1.9)	1 (1.9)
z-score INeS	mean (SD) (min–max)	−0.32 (1.06) (−3.27–+1.88)	−0.79 (1.28) (−3.24–+1.74)
<10th centile INeS	*n* (%)	11 (20.4)	20 (38.5)
>90th centile INeS	*n* (%)	4 (7.4)	3 (5.8)
**Birth length** **			
cm	mean (SD) (min–max)	36.7 (3.1) (30.4–43.4)	37.8 (2.9) (31.0–45.9)
z-score Int21s	mean (SD) (min–max)	−0.98 (0.89) (−2.89–+0.88)	−1.33 (1.35) (−4.04–+2.01)
<10th centile Int21st	*n* (%)	15 (34.9)	22 (50.0)
>90th centile Int21st	*n* (%)	0 (0.0)	1 (2.3)
z-score INeS	mean (SD) (min–max)	−0.56 (0.99) (−2.68–+1.60)	−0.97 (1.42) (−3.47–+2.33)
<10th centile INeS	*n* (%)	11 (25.0)	19 (43.2)
>90th centile INeS	*n* (%)	1 (2.3)	3 (6.8)
**Birth weight**			
g	mean (SD) (min–max)	1129 (321) (570–2040)	1196 (304) (520–1900)
z-score Int21s	mean (SD) (min–max)	−0.67 (1.17) (−2.90–+1.56)	−1.12 (1.20) (−3.89–+1.36)
<10th centile Int21st (SGA)	*n* (%)	21 (35.0)	28 (46.7)
>90th centile Int21st (LGA)	*n* (%)	2 (3.3)	1 (1.7)
z-score INeS	mean (SD) (min–max)	−0.31 (1.12) (−2.36–+1.93)	−0.80 (1.16) (−3.15–+1.19)
<10th centile INeS (SGA)	*n* (%)	14 (23.0)	23 (38.3)
>90th centile INeS (LGA)	*n* (%)	4 (6.6)	0 (0.0)

BF-arm: preterm infants randomized on adjustable fortification with bovine milk based supplements. DF-arm: preterm infants randomized on adjustable fortification with donkey milk based supplements. IQR: interquartile range. SD: standard deviation. GA: gestational age. SGA: small for gestational age (birthweight < 10th centile). LGA: large for gestational age (birthweight > 90th centile). *n* = 59 (BF-arm = 35, DF-arm = 24). * *n* = 106 (BF-arm = 54, DF-arm = 52). ** *n* = 88 (BF-arm = 44, DF-arm = 44).

**Table 2:** T-test results: means z-score [CI(95%)] per arm, difference between donkey milk-based fortifier (DF-) and bovine milk-based fortifier (BF)-arm [CI(95%)], and *p*-value of difference.

	BF-Arm		DF-Arm		DF-BF	
	*n*	Mean [CI(95%)]	*n*	Mean [CI(95%)]	Mean [CI(95%)]	*p*
Head circumference	61	−0.066 [−0.372; +0.239]	59	−0.132 [−0.434; +0.169]	−0.066 [−0.491; +0.359]	0.758
Length	58	−1.530 [−1.843; −1.216]	58	−1.604 [−1.900; −1.309]	−0.075 [−0.501; +0.351]	0.729
Weight	60	−0.955 [−1.230; −0.681]	60	−0.997 [−1.228; −0.716]	−0.041 [−0.430; +0.348]	0.833

BF-arm: preterm infants randomized on adjustable fortification with bovine milk-based supplements. DF-arm: preterm infants randomized on adjustable fortification with donkey milk-based supplements. CI: confidence interval.

**Table 3:** General linear model results: LS-means z-score [CI(95%)] per arm, difference between DF- and BF-arm [CI(95%)] and *p*-value of difference. For each auxological variable, the estimates of model with birth z‑score according to neonatal Intergrowth21st and INeS charts are reported.

	BF-Arm	DF-Arm	DF-BF	*p*
Head circumference				
*Int21st*	−0.101 [−0.412; +0.210]	−0.104 [−0.430; +0.221]	−0.003 [−0.326; +0.319]	0.986
*INeS*	−0.136 [−0.437; +0.165]	−0.132 [−0.449; +0.184]	+0.004 [−0.311; +0.319]	0.985
Length/Height				
*Int21st*	−1.786 [−2.141; −1.431]	−1.658 [−2.016; −1.300]	+0.128 [−0.230; +0.478]	0.565
*INeS*	−1.846 [−2.192; −1.500]	−1.722 [−2.072; −1.372]	+0.124 [−0.224; +0.481]	0.551
Weight				
*Int21st*	−1.050 [−1.349; −0.751]	−0.983 [−1.289; −0.677]	+0.067 [−0.237; +0.372]	0.716
*INeS*	−1.057 [−1.360; −0.755]	−0.995 [−1.310; −0.679]	+0.063 [−0.246; +0.372]	0.738

BF-arm: preterm infants randomized on adjustable fortification with bovine milk based supplements. DF-arm: preterm infants randomized on adjustable fortification with donkey milk based supplements. CI: confidence interval. LS-means: Least squares means.

**Table 4:** General linear model results: effect estimate of birth z-score, gestational age and population on auxological variables at 18 months of life. LS-means [CI (95%)].

	Head Circumference	Length	Weight
**BW at birth**			
Intergrowth21st	+0.597 [+0.422; +0.771]	+0.537 [+0.330; +0.744]	+0.449 [+0.298; +0.601]
INeS	+0.568 [+0.414; +0.723]	+0.521 [+0.336; +0.706]	+0.461 [+0.288; +0.633]
**GA at birth**			
Intergrowth21st	+0.021 [−0.068; +0.109]	+0.176 [+0.076; +0.277]	+0.116 [+0.033; +0.198]
INeS	+0.049 [−0.035; +0.132]	+0.218 [+0.123; +0.314]	+0.139 [+0.057; +0.220]
**POP: <32 weeks vs. ≥32 weeks**			
Intergrowth21st	−0.102 [−0.647; +0.442]	+0.058 [−0.566; +0.683]	+0.163 [−0.352; +0.679]
INeS	+0.009 [−0.506; +0.525]	+0.206 [−0.385; +0.796]	+0.157 [−0.375; +0.690]

CI: confidence interval. LS-means: Least squares means.
